# Deep Learning Model for Classifying Metastatic Epidural Spinal Cord Compression on MRI

**DOI:** 10.3389/fonc.2022.849447

**Published:** 2022-05-04

**Authors:** James Thomas Patrick Decourcy Hallinan, Lei Zhu, Wenqiao Zhang, Desmond Shi Wei Lim, Sangeetha Baskar, Xi Zhen Low, Kuan Yuen Yeong, Ee Chin Teo, Nesaretnam Barr Kumarakulasinghe, Qai Ven Yap, Yiong Huak Chan, Shuxun Lin, Jiong Hao Tan, Naresh Kumar, Balamurugan A. Vellayappan, Beng Chin Ooi, Swee Tian Quek, Andrew Makmur

**Affiliations:** ^1^ Department of Diagnostic Imaging, National University Hospital, Singapore, Singapore; ^2^ Department of Diagnostic Radiology, Yong Loo Lin School of Medicine, National University of Singapore, Singapore, Singapore; ^3^ NUS Graduate School, Integrative Sciences and Engineering Programme, National University of Singapore, Singapore, Singapore; ^4^ Department of Computer Science, School of Computing, National University of Singapore, Singapore, Singapore; ^5^ Department of Radiology, Ng Teng Fong General Hospital, Singapore, Singapore; ^6^ National University Cancer Institute, NUH Medical Centre (NUHMC), Singapore, Singapore; ^7^ Biostatistics Unit, Yong Loo Lin School of Medicine, Singapore, Singapore; ^8^ Division of Spine Surgery, Department of Orthopaedic Surgery, Ng Teng Fong General Hospital, Singapore, Singapore; ^9^ University Spine Centre, Department of Orthopaedic Surgery, National University Health System, Singapore, Singapore; ^10^ Department of Radiation Oncology, National University Cancer Institute Singapore, National University Hospital, Singapore, Singapore

**Keywords:** deep learning model, metastatic epidural spinal cord compression, MRI, Bilsky classification, spinal metastasis classification, spinal metastatic disease, epidural spinal cord compression

## Abstract

**Background:**

Metastatic epidural spinal cord compression (MESCC) is a devastating complication of advanced cancer. A deep learning (DL) model for automated MESCC classification on MRI could aid earlier diagnosis and referral.

**Purpose:**

To develop a DL model for automated classification of MESCC on MRI.

**Materials and Methods:**

Patients with known MESCC diagnosed on MRI between September 2007 and September 2017 were eligible. MRI studies with instrumentation, suboptimal image quality, and non-thoracic regions were excluded. Axial T2-weighted images were utilized. The internal dataset split was 82% and 18% for training/validation and test sets, respectively. External testing was also performed. Internal training/validation data were labeled using the Bilsky MESCC classification by a musculoskeletal radiologist (10-year experience) and a neuroradiologist (5-year experience). These labels were used to train a DL model utilizing a prototypical convolutional neural network. Internal and external test sets were labeled by the musculoskeletal radiologist as the reference standard. For assessment of DL model performance and interobserver variability, test sets were labeled independently by the neuroradiologist (5-year experience), a spine surgeon (5-year experience), and a radiation oncologist (11-year experience). Inter-rater agreement (Gwet’s kappa) and sensitivity/specificity were calculated.

**Results:**

Overall, 215 MRI spine studies were analyzed [164 patients, mean age = 62 ± 12(SD)] with 177 (82%) for training/validation and 38 (18%) for internal testing. For internal testing, the DL model and specialists all showed almost perfect agreement (kappas = 0.92–0.98, p < 0.001) for dichotomous Bilsky classification (low versus high grade) compared to the reference standard. Similar performance was seen for external testing on a set of 32 MRI spines with the DL model and specialists all showing almost perfect agreement (kappas = 0.94–0.95, p < 0.001) compared to the reference standard.

**Conclusion:**

A DL model showed comparable agreement to a subspecialist radiologist and clinical specialists for the classification of malignant epidural spinal cord compression and could optimize earlier diagnosis and surgical referral.

## Introduction

Spinal metastases are common and seen in up to 40% of cancer patients. Up to 20% of these patients develop complications including spinal cord compression, which can lead to permanent neurological dysfunction if treatment is delayed. With the development of more effective systemic therapy (such as targeted and immunotherapy), the survival of patients with metastatic cancer has increased, and consequently, the incidence of spinal metastases is expected to rise (1–3).

Suspicion for spinal metastases begins in the clinic, as greater than 85% of patients present with back pain. Imaging is then required to confirm the presence of spinal metastases and the associated complications. MRI is the most accurate modality due to improved soft-tissue resolution, which allows assessment of the extent of metastatic bony involvement, compression fractures, and the presence of metastatic epidural spinal cord compression (MESCC) (4).

The degree of MESCC is assessed on axial T2-weighted (T2W) MR images using a six-point grading scale developed by the Spine Oncology Study Group (SOSG), commonly referred to as the Bilsky grading scale (5). Low-grade disease (Bilsky 0, 1a, and 1b) can be considered for initial radiotherapy (including stereotactic body radiotherapy (SBRT)/stereotactic radiosurgery), whereas higher-grade disease (Bilsky 1c, 2, and 3) should be considered for surgical decompression followed by radiotherapy (6). MESCC requires urgent treatment to prevent permanent neurological injury, but significant delays in management have been reported. A study by van Tol et al. (2021) showed median delays of 21.5, 7, and 8 days for the diagnosis, referral, and treatment of MESCC, respectively (7).

A deep learning (DL) model to automatically detect and classify low- versus high-grade Bilsky MESCC on MRI could alert the radiologist and clinical teams, ensuring prompt reporting and appropriate referral. This is important to prevent poor functional outcomes and increased requirements of healthcare resources (8). Automated tools for detecting urgent findings on MRI are important due to increasing demand for the modality, while faced with a shortage of radiologists (9). In the United Kingdom, 3.4 million MRI studies are reported every year, and patients can wait over 30 days for a report (10, 11). Even for emergent indications including suspected MESCC where reporting should be performed within hours, more than a third of reports were provided greater than 48 h later at one healthcare trust (10, 12).

Prior DL in spine MRI has shown promise, especially with the use of convolutional neural networks (CNNs), which can automatically learn representative features from images to perform classification tasks. Most recently, several teams have developed DL models for the automated classification of degenerative narrowing in the lumbar spine (13, 14) or adjacent segment disease along the cervical spine (15). DL for spinal metastases on advanced imaging, including MRI, is still in the preliminary phase. A study by Wang et al. (2017) showed the feasibility of automated spinal metastatic disease detection on MRI using a small set of 26 patients (16). The group achieved a true positive rate of 90% with a false-positive rate of up to 0.4 per case. DL for the detection of spinal metastases on CT has also shown promise for quantifying metastatic bone disease burden (17). Currently, to our knowledge, no DL model has been developed to assess MESCC on MRI.

The aim of this study was to train a DL model for the automated Bilsky classification of MESCC using axial T2W MRI. This could aid earlier diagnosis of MESCC and identify suitable candidates for radiotherapy versus emergent surgical decompression. Once trained, the performance of the DL model was compared with that of a radiation oncologist, spine oncology surgeon, and subspecialty radiologist, on an internal test set. The DL model performance and generalizability were also assessed on an external test set.

## Materials and Methods

This study was approved by our institutional review board and compliant with the Health Insurance Portability and Accountability Act (HIPAA). A waiver of consent was granted due to the retrospective nature of the study and the minimal risk involved.

### Dataset Preparation

Retrospective, manual extraction, and anonymization of MRI spines from patients with known vertebral metastatic disease and thoracic MESCC were done over a 10-year period from September 2007 to September 2017 at the National University Hospital, Singapore. Adult patients (≥18 years) were included with a selection of studies across different MRI scanners (GE and Siemens 1.5- and 3.0-T platforms). A heterogeneous training dataset obtained using a range of MRI platforms and T2W parameters was used to prevent overfitting and provide a more generalizable DL algorithm. MRI spines with instrumentation, suboptimal image quality (e.g., motion and cerebrospinal fluid flow artifacts), and non-thoracic spine regions were excluded. Axial T2W DICOM images were utilized. **Supplementary Table 1** provides details on the MRI scanners and T2W sequence parameters.

The dataset at the National University Hospital, Singapore, was assigned as the internal dataset and was randomly split into 82% and 18% for the training/validation and test sets, respectively. This is an acceptable split for DL datasets (18).

A dataset of MRI spine studies from patients with known metastatic disease and MESCC was also obtained for external testing from Ng Teng Fong General Hospital (Siemens 1.5-T MRI platform). The inclusion and exclusion criteria were identical to the internal dataset. The MRI spines were obtained over a 5-year period from September 2015 to September 2020, encompassing anonymized axial T2W DICOM images. No further training was performed on this dataset.

### Dataset Labelling

Internal training data were manually labeled by two board-certified radiologists with sub-specialization in musculoskeletal radiology (JH; 10-year experience) and neuroradiology (AM; 5-year experience). Each radiologist labeled at least 100 MRI thoracic spine studies independently. With the use of an open-source annotation software (LabelImg, https://github.com/tzutalin/labelImg), bounding boxes were drawn to segment the region of interest (ROI) around the spinal canal along the thoracic spine (C7–T1 through to the conus at T12–L3). A bounding box was placed on each axial T2W image.

**Figure 1:**
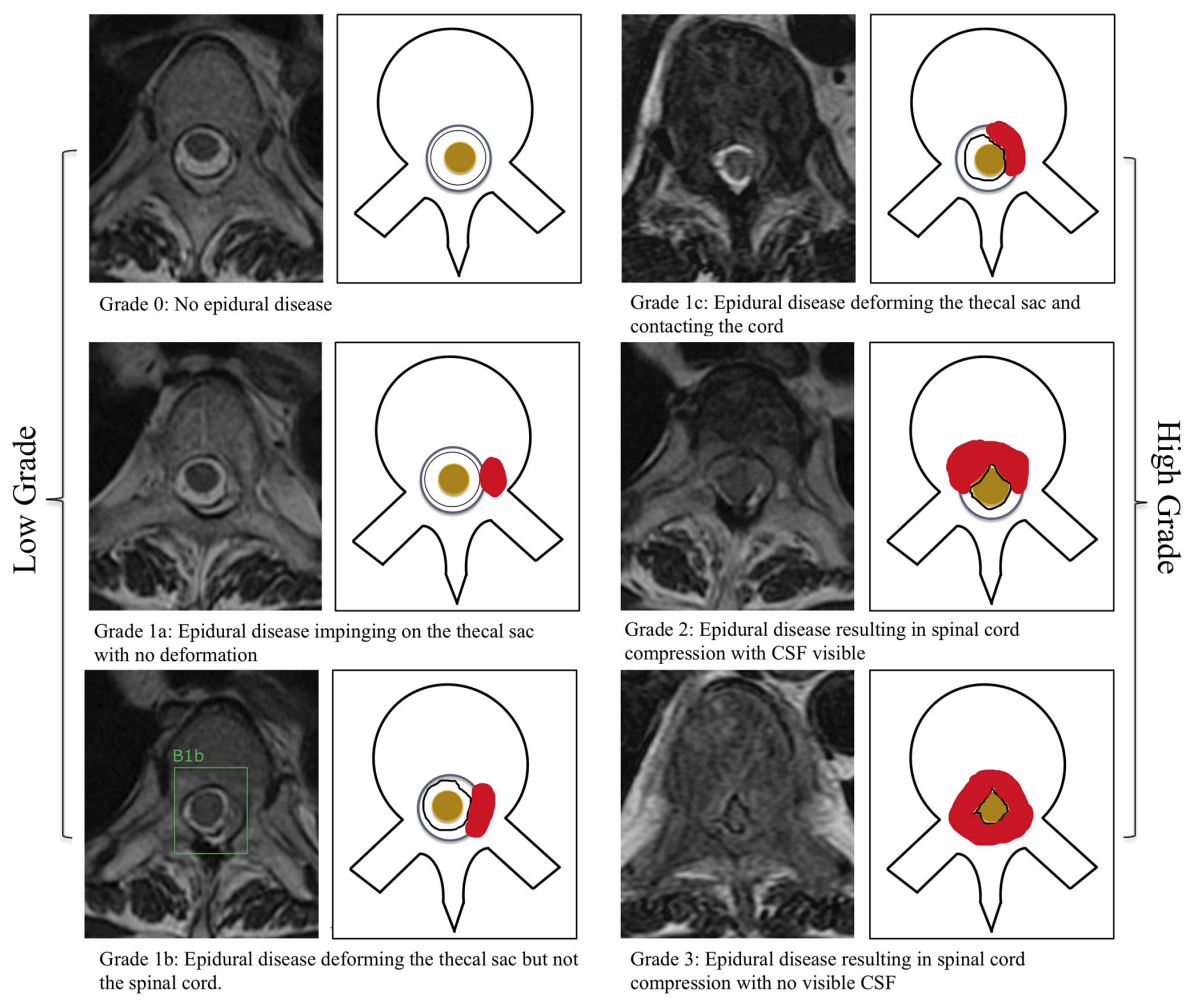
Bilsky classification of metastatic epidural spinal cord compression on MRI of the thoracic spine. Axial T2-weighted (repetition time ms/echo time ms, 5,300/100) images were used. Training of the deep learning model was performed by a radiologist by placing a bounding box around the region of interest at each T2-weighted image. A bounding box example is included for a low-grade Bilsky 1b lesion (1b). CSF, cerebrospinal fluid.

When drawing each bounding box, the annotating radiologist classified the MESCC using the Bilsky classification (4). This grading scheme consists of six classifications with grades 0, 1a, and 1b amenable to radiotherapy and grades 1c, 2, and 3 more likely to require surgical decompression. A visual scale was provided to all annotating readers (**Figure 1**). Degenerative changes (disk bulges and ligamentum flavum redundancy) leading to moderate-to-severe spinal canal stenosis were labeled by the annotating radiologists and excluded from further analysis (19, 20).

The internal and external test sets were labeled using the same visual scale by the musculoskeletal radiologist (JH) with 10-year experience and served as the reference standard. For comparison with the DL model and to assess interobserver variability, the internal and external test sets were also labeled independently by a subspecialist neuroradiologist (AM; 5-year experience), a spine oncology surgeon (JT; 5-year experience), and a radiation oncologist (BV; 11-year experience). The specialist readers were blinded to the reference standard.

### Deep Learning Model Development

A convolutional prototypical network is a newly proposed neural network architecture for robust image classification with cluster assumption (21). Specifically, it is assumed that there exists an embedding space in which data points cluster around a single prototype representation for each class. Different types of loss functions are proposed for the training of the network with the general stochastic gradient descent method (22). Several studies have demonstrated the robustness of convolutional prototypical networks towards data scarcity and class imbalance problems, which have also led to more compact and discriminative features in the embedding space (21, 22).

In this paper, a convolutional prototypical network was trained with ResNet50 as its backbone to project ROI images into a high-dimensional embedding space (23). The Apache SINGA (24) platform was adopted for efficient training of the deep network, and MLCask (25), an efficient data analytics pipeline management system, was adopted to facilitate managing different versions of the developed pipelines. We used the output from the global average layer of ResNet50 as the feature representation for each image in the embedding space. A class prototype was assigned for each Bilsky score in the embedding space. The prediction probability of a data point was calculated for each class *via* a SoftMax over the negative distance to the class prototypes. The network was trained with a cross-entropy loss on the prediction probability using a standard SGD optimizer, and a compact regularization was introduced to further minimize the distance between the data points and their corresponding class prototypes. Simultaneously, the virtual adversarial loss was introduced to ensure our model makes consistent predictions around the neighborhood of each data point with adversarial local perturbation (26). An ablation study was also conducted to demonstrate the effectiveness of the virtual adversarial loss and the compact regularization loss. The ablation study details are included as the Supplementary Material, and **Supplementary Table 2** shows the ablation study results. The Supplementary Material including **Supplementary Table 3** has also been provided to compare our developed model with both the standard ResNet50 and the plain convolutional prototypical network.

For inference, the first step is to extract the ROI of an input image. The extracted ROI is then projected into the embedding space. Finally, the label of the input image is predicted as the label of its nearest class prototype in the embedding space (**Supplementary Figure 1**). The DL model (SpineAI@NUHS-NUS) code is at https://github.com/NUHS-NUS-SpineAI/SpineAI-Bilsky-Grading. **Supplementary Figure 2** shows a flow chart of the developed DL model in a clinical setting.

### Statistical Analysis

All analyses were performed using Stata version 16 (StataCorp, College Station, TX, USA) with statistical significance set at 2-sided p < 0.05. Postulating that a kappa of 0.9 is to be anticipated, at least 138 samples (MRI studies) were required to provide a 95% CI width of 0.1. Over the 10-year study period, 174 subjects with 239 MRI studies were collected, which was sufficient for the analysis. Descriptive statistics for continuous variables were presented as mean ± SD (range) and n (%) for categorical variables. Inter-rater agreement using dichotomous (low-grade versus high-grade) Bilsky classification was assessed using Gwet’s kappa to account for the paradox effect of a high percentage of normal classification (27). Sensitivity and specificity were also presented for dichotomous Bilsky gradings only. Sensitivity is the percentage of high-grade Bilsky classifications that are correctly identified by the DL model and specialist readers, whereas specificity is the percentage of low-grade Bilsky classifications that are correctly identified by the DL model and specialist readers.

Levels of agreement were defined for Gwet’s kappa: <0 = poor, 0–0.2 = slight, 0.21–0.4 = fair, 0.41–0.6 = moderate, 0.61–0.8 = substantial, and 0.81–1 = almost-perfect agreement (28). Also, 95% CIs were calculated.

## Results

### Patient Characteristics in Datasets

Data collection over the 10-year study period identified 174 patients with 239 MRI spines for analysis. Of these, 24 MRI spines from 10 patients were excluded due to instrumentation (4 MRI spines), suboptimal image quality (2 MRI spines), or non-thoracic spine MRI (18 MRI spines). A total of 164 patients encompassing 215 MRI thoracic spines were evaluated. Overall, the mean age of all 164 patients was 62 ± 12 (SD) (range: 18–93 years). The patient group was predominantly male (91/164 patients, 55.4%), with breast and lung being the most common primary cancers (63/164 patients, 38.4%). There was a wide range of sites of MESCC along the thoracic region, with a predominance of disease in the semirigid thoracic region between T3 and T10 (73/164 patients, 44.5%). The patient demographics, cancer subtypes, and MESCC distribution along the thoracic region for the training and test sets are displayed in **Table 1**.

**Table 1:** Patient demographics and clinical characteristics for the internal and external test sets.

Characteristics	Internal training set (n = 129)	Internal test set (n = 35)	External test set (n = 32)
Age (years)^*^	61 ± 13 (18–93)	61 ± 12 (39–87)	60 ± 13 (19–85)
Women	55 (42.6)	18 (51.4)	12 (37.5)
Men	74 (57.4)	17 (48.6)	20 (62.5)
Ethnicity			
Chinese	93 (72.1)	28 (80)	23 (71.9)
Malay	21 (16.3)	3 (8.6)	7 (21.9)
Indian	7 (5.4)	2 (5.7)	0 (0)
Others	8 (6.2)	2 (5.7)	2 (6.2)
Cancer subtype			
Breast	23 (17.8)	8 (22.9)	3 (9.4)
Lung	21 (16.3)	11 (31.4)	13 (40.6)
Prostate	19 (14.7)	5 (14.3)	4 (12.5)
Colon	15 (11.6)	3 (8.6)	3 (9.4)
Renal cell carcinoma	10 (7.8)	2 (5.7)	1 (3.1)
Nasopharyngeal carcinoma	9 (7)	3 (8.6)	1 (3.1)
Others	32 (24.8)	3 (8.6)	7 (21.9)
No. of MRI thoracic spines	177/215 (82.3)	38/215 (17.6)	32
MESCC location			
Diffuse thoracic^#^	30 (23.3)	8 (22.9)	3 (9.4)
C7–T2	13 (10.1)	3 (8.6)	6 (18.8)
T3–T10	55 (42.6)	18 (51.4)	15 (46.9)
T11–L3	31 (24.0)	6 (17.1)	8 (25)

MESCC, malignant epidural spinal cord compression.

^*^Values are mean ± SD (range) for numerical variables and n (%) for categorical variables.

^#^Two or more sites of thoracic epidural disease.

**Figure 2:**
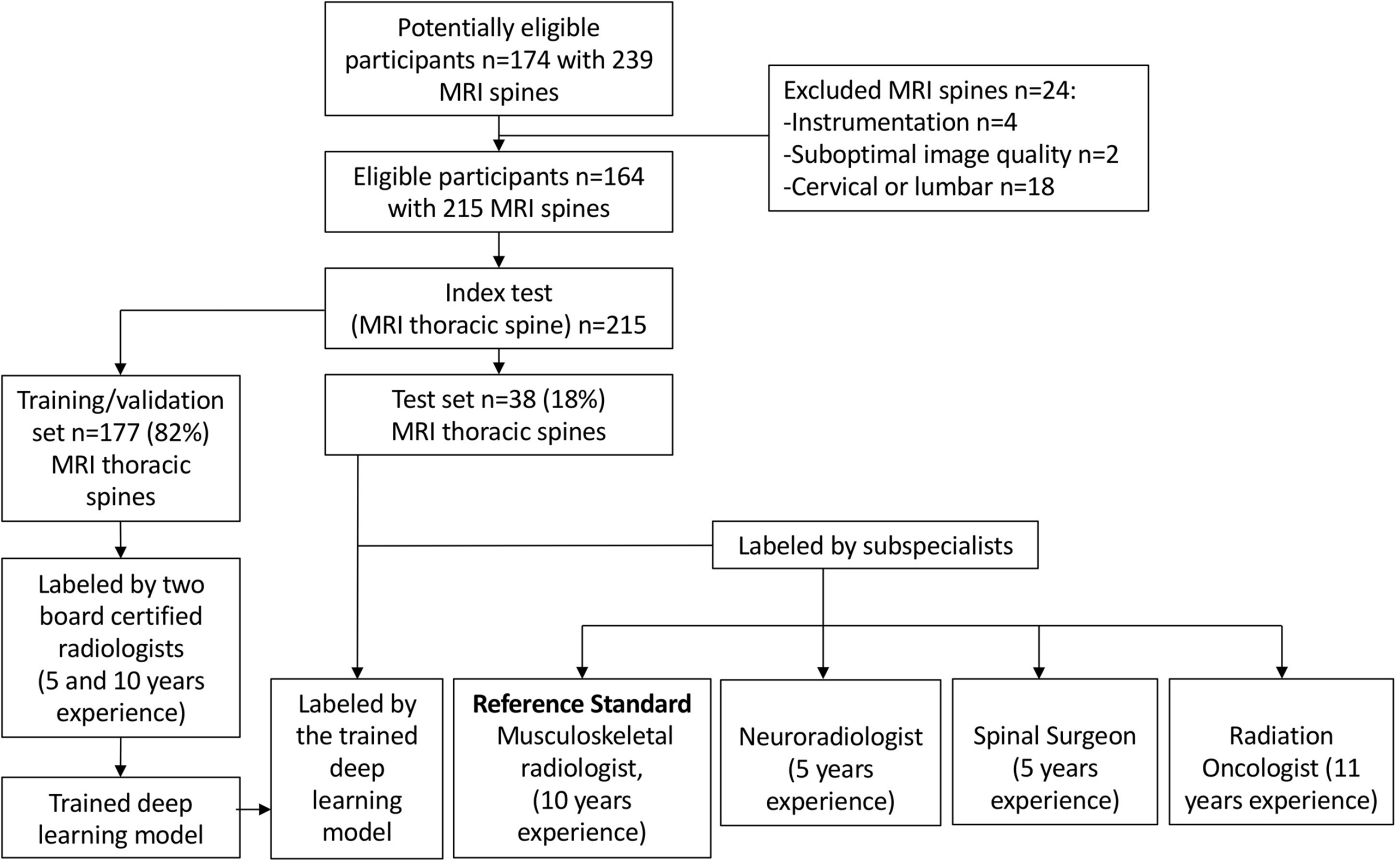
Flow chart of the study design for the internal training/validation and test sets. The deep learning model performance was compared with an expert musculoskeletal radiologist (reference standard) and three specialist readers.

The internal dataset of 215 MRI spines was randomly split into 177 (82%) studies for training/validation and 38 (18%) studies for internal testing. A flow chart of the internal dataset study design is provided in **Figure 2**.

For the external dataset, 32 patients with 32 MRI spines covering the thoracic region were available for external testing. Overall, the mean age of the 32 patients was 60 ± 13 (SD) (range: 19–85 years). Similar to the internal dataset, the patient group had a predominance of men (20/32 patients, 62.5%), with the lung being the most common primary cancer (13/32 patients, 40.6%).

### Reference Standard

The number of ROIs and the corresponding Bilsky classifications in the internal training and test sets, and external test sets are highlighted in **Table 2**. In the internal training/validation set, high-grade Bilsky classification (1c/2/3) accounted for 462/5,863 ROIs (7.9%) with a predominance of low-grade Bilsky classification (0/1a/1b) at the remaining 5,401/5,863 ROIs (92.1%). In the internal test set, high-grade Bilsky classification (1c/2/3) accounted for 84/1,066 ROIs (7.9%) with a predominance of low-grade Bilsky classification (0/1a/1b) at the remaining 982/1,066 ROIs (92.1%). In comparison, for the external test set, there was a greater proportion of high-grade Bilsky classification (169/754 ROIs, 22.4%) and a reduced predominance of low-grade Bilsky classification (585/754 ROIs, 77.6%). The greater proportion of higher-grade Bilsky classification in the external test set was likely due to more targeted axial T2W images at the sites of MESCC.

**Table 2:** Reference standards for the internal (training and test) and external (test) sets showing the number of Bilsky MESCC grades.

Bilsky MESCC grade	Internal training/validation set	Internal test set	External test set
0	4,508 (76.9)	849 (79.6)	454 (60.2)
1a	424 (7.2)	82 (7.7)	48 (6.4)
1b	469 (8.0)	51 (4.8)	83 (11)
1c	216 (3.7)	35 (3.3)	51 (6.7)
2	105 (1.8)	26 (2.4)	39 (5.2)
3	141 (2.4)	23 (2.2)	79 (10.5)
**Total**	5,863	1,066	754

Values are n (%). A region of interest (bounding box) for Bilsky grade was drawn at each axial T2-weighted image.

MESCC, malignant epidural spinal cord compression.

### Internal Test Set Region of Interest Classification

For the internal dataset, there was almost perfect agreement between the reference standard for dichotomous Bilsky classification and the DL model and all specialist readers, with kappas ranging from 0.92 to 0.98, all p < 0.001 (**Table 3**). A kappa of 0.98 (95% CI = 0.97–0.99, p < 0.001) for the spine surgeon was the highest, with similar kappas of 0.97 (95% CI = 0.96–0.98, p < 0.001) and 0.96 (95% CI = 0.95–0.98, p < 0.001) for the radiation oncologist and neuroradiologist, respectively. DL model kappa of 0.92 (95% CI = 0.91–0.94, p < 0.001) was slightly lower compared to that of the specialist readers.

**Table 3:** Internal and external test set classifications using dichotomous Bilsky gradings (low versus high grade) on MRI.

Reader	Internal test set		External test set	
	Kappa (95% CI)	p-Value	Kappa (95% CI)	p-Value
DL model	0.92 (0.91–0.94)	<0.001	0.94 (0.92–0.96)	<0.001
Neuroradiologist	0.96 (0.95–0.98)	<0.001	0.95 (0.93–0.97)	<0.001
Radiation oncologist	0.97 (0.96–0.98)	<0.001	0.94 (0.92–0.96)	<0.001
Spine surgeon	0.98 (0.97–0.99)	<0.001	0.94 (0.91–0.96)	<0.001

Gwet’s kappa was used.

DL, deep learning model.

The sensitivity for the DL model (97.6%, 95% CI = 91.7%–99.7%) was the highest for the internal dataset, and this was significantly higher compared to both the neuroradiologist (84.5%, 95% CI = 75.0%–91.5%) and spine surgeon (79.8%, 95% CI = 69.6%–87.7%), p = 0.003 and p < 0.001, respectively (**Table 4** and confusion matrix in **Supplementary Table 4**). High specificities (range = 93.6%–99.5%) were seen for the DL model and specialists. The spine surgeon had a specificity of 99.5% (95% CI = 98.8%–99.8%), which was significantly higher than the DL model, neuroradiologist, and radiation oncologist, with specificities of 93.6% (95% CI = 91.9%–95.0%), 98.1% (95% CI 97.0%–98.8%), and 97.9% (95% CI = 96.7%–98.7%), p < 0.001, p = 0.004, and p = 0.002, respectively.

**Table 4:** Internal and external test set sensitivity and specificity for the deep learning model and specialist readers using dichotomous Bilsky gradings (low versus high grade) on MRI.

Reader	Internal test set		External test set	
	Sens (95% CI)	Spec (95% CI)	Sens (95% CI)	Spec (95% CI)
DL model	97.6 (91.7–99.7)	93.6 (91.9–95.0)	89.9 (84.4–94.0)	98.1 (96.7–99.1)
Neuroradiologist	84.5 (75.0–91.5)	98.1 (97.0–98.8)	92.9 (87.9–96.2)	97.9 (96.4–98.9)
Radiation oncologist	94.0 (86.7–98.0)	97.9 (96.7–98.7)	88.8 (83.0–93.1)	98.5 (97.1–99.3)
Spine surgeon	79.8 (69.6–87.7)	99.5 (98.8–99.8)	83.4 (77.0–88.7)	99.3 (98.3–99.8)

DL, deep learning model; Sens, sensitivity; Spec, specificity.

### External Test Set Region of Interest Classification

For the external dataset, the DL model and all the specialist readers also had almost perfect agreement (kappas 0.94–0.95, all p < 0.001) compared to the reference standard for dichotomous Bilsky classification (**Table 3**). The neuroradiologist kappa of 0.95 (95% CI = 0.93–0.97, p < 0.001) was only slightly higher compared to the rest, with similar kappas of 0.94 (95% CI = 0.92–0.96, p < 0.001), 0.94 (95% CI = 0.92–0.96, p < 0.001), and 0.94 (95% CI = 0.91–0.96, p < 0.001) for the DL model, radiation oncologist, and spine surgeon, respectively.

The sensitivity for the DL model on the external dataset was 89.9% (95% CI = 84.4%–94.0%), and this was not significantly different from the other readers, including the neuroradiologist with the highest sensitivity of 92.9% (95% CI = 87.9%–96.2%), all p > 0.05 (**Table 4** and confusion matrix in **Supplementary Table 5**). The neuroradiologist had no significantly higher sensitivity compared to the other readers, all p > 0.05. The spine surgeon had a specificity of 99.3% (95% CI = 98.3%–99.8%), which was significantly higher than the specificity of the neuroradiologist at 97.9% (95% CI 96.4%–98.9%), p = 0.042.

## Discussion

MRI is an essential tool in the assessment of MESCC, which is a potentially devastating complication of advanced cancer. Bilsky et al. (2010) developed an MRI classification for MESCC that aimed to improve communication between specialists and aid decision making for initial radiotherapy versus expedited surgical decompression. In our study, we trained a DL model for automated Bilsky MESCC classification on thoracic spine MRI using manual radiologist labels. On an internal test set, the DL model showed almost-perfect agreement (κ = 0.92, p < 0.001) for dichotomous Bilsky classification (low grade versus high grade), similar to specialist readers (κ = 0.96–0.98, all p < 0.001), which included a radiation oncologist, a neuroradiologist, and a spine surgeon. In a further step, external testing of the DL model was performed on a dataset from a different institution to assess generalizability. For the external dataset, the DL model and all the specialist readers also had almost perfect agreement (kappas 0.94–0.95, all p < 0.001) for dichotomous Bilsky classification.

DL is already being used in spine diseases to aid in the diagnosis of spinal stenosis on MRI spines, surgical planning, and prediction of outcomes in patients with spinal metastases (8, 29). DL in spinal oncology imaging is limited with most researchers focusing on the detection of metastases (30), or automated spinal cord segmentation as an organ at risk for radiotherapy planning (31). Average Dice similarity coefficients for spinal cord segmentation are as high as 0.9 for automated lung cancer radiotherapy planning using DL on CT studies (32, 33). Automated detection of spinal cord compression on MRI has currently only been assessed in the cervical spine. Merali et al. (2021) developed a DL model for degenerative cervical spinal cord compression on MRI using 201 patients from a surgical database (34). Their DL model had an overall AUC of 0.94 with a sensitivity of 0.88 and specificity of 0.89.

To our knowledge, no team has currently looked at the automated prediction of metastatic epidural spinal cord compression on MRI, which is a medical emergency. The current National Institute for Health and Care Excellence (NICE) guidelines state that metastatic epidural spinal cord compression should be treated as soon as possible, ideally within 24 h, to prevent irreversible neurological dysfunction (35). Our MRI Bilsky grading prediction model could improve the imaging and clinical workflow of patients with spinal metastases. MRI studies with MRI studies with high-grade Bilsky disease could be triaged for urgent radiologist review, with the radiology reporting augmented by an automated selection of key images at the sites of the highest-grade Bilsky lesions and spinal cord compression. These key images could also be circulated to an on-demand spine oncology multidisciplinary team (spine surgeons, oncologists, and radiation oncologists) for more streamlined decision making and appropriate referral. It should be emphasized that the treatment of MESCC is not just dependent on imaging but is also heavily weighted on clinical presentation, e.g., myelopathy, weakness, and loss of bowel and bladder function. Individuals can present with high-grade Bilsky scores and not be suitable surgical candidates. Further work using our Bilsky prediction model could involve combining imaging data with clinical information (e.g., age, cancer subtype, and degree of neurological impairment) to improve the selection of patients for more aggressive management including surgery and/or SBRT (21, 36). Our DL model is focused on Bilsky classification and currently does not have the ability to segment or outline tumors. DL auto-segmentation of tumors in MR images could optimize and reduce the time taken for radiotherapy planning (32). Future research will focus on developing a DL model for this application, which will be especially useful for SBRT.

Our study has limitations. First, we utilized axial T2W images along the thoracic region, which was recommended as the most accurate method for MESCC classification on MRI in the study by Bilsky et al. (2010) (4). In further studies, we could enhance the model performance for the detection and classification of MESCC by combining multiple MRI sequences, including sagittal T2W and gadolinium-enhanced T1-weighted axial and sagittal image sets. Second, we chose to use dichotomous Bilsky classification (low grade vs. high grade) with the inclusion of Bilsky 1c under high-grade disease. This is controversial, as patients with Bilsky 1c are unlikely to have neurological deficits requiring urgent surgical treatment. However, for the purpose of treatment triaging, we decided to be more conservative and classify 1c under high grade. Third, the reference standard was a single expert musculoskeletal radiologist who reviewed the test set independently from the other three specialist readers. No consensus labeling was performed for the readers, as this may have been biased toward the expert. Fourth, the test sets were only assessed by specialist readers to ensure the most rigorous comparison with the DL model. Assessment by less experienced readers (e.g., radiology or surgical trainees) was not analyzed but could be performed through further studies that include the use of semi-supervised reporting augmentation by the DL model. Finally, labeling of images for model development was a labor-intensive manual process (highly supervised). This was believed to be the most accurate method for training the model but potentially limited the number of MRI studies that could be used for training. Alternatively, future larger datasets could utilize semi-supervised learning, which can leverage unlabeled data to boost the DL model performance and reduce the data annotation burden (37–39). Future work could also utilize additional external datasets to ensure the DL model is not overfitted to our institution data and is generalizable to new, unseen data.

In conclusion, we demonstrated that our DL model is reliable and may be used to automatically assess the Bilsky classification of metastatic epidural spinal cord compression on thoracic spine MRI. In clinical practice, the early diagnosis of spinal cord compression is important to prevent permanent neurological dysfunction (40). The DL model could be used to triage MRI scans for urgent reporting, augment non-sub-specialized radiologists when they report out of hours, and improve the communication and referral pathways between specialties including oncology, radiation oncology, and surgery. Finally, the proposed framework, which makes use of Apache SINGA (24) for distributed training, has been integrated into our MLCask (25) system for handling healthcare images and analytics.

## Data Availability Statement

The original contributions presented in the study are included in the article/**Supplementary Material**. Further inquiries can be directed to the corresponding author.

## Author Contributions

Conception, methodology, data curation, supervision, visualization, and writing: JH, LZ, WZ, DL, SB, XL, ET, NBK, QY, YC, JT, NK, BV, BO, SQ, and AM. Investigation and project administration: JH, LZ, WZ, DL, SB, XL, ET, NBK, QY, YC, SL, JT, NK, BV, BO, and AM. Resources and software: JH, LZ, WZ, DL, KY, QY, YC, SL, NK, BV, BO, SQ, and AM. Formal analysis and validation: JH, LZ, WZ, DL, KY, NBK, QY, YC, SL, JT, NK, BV, BO, and SQ.

## Funding

1) Direct funding from MOH/NMRC: this research is supported by the Singapore Ministry of Health’s National Medical Research Council under its NMRC Clinician-scientist individual research grant, new investigator grant (CS-IRG NIG); Grant Title: Deep learning pathway for the management of spine metastases (CNIG20nov-0011, MOH-000725). 2) NCIS Centre Grant Seed Funding Program (December 2020 Grant Call); Grant Title: Artificial intelligence for the management of vertebral metastases.

## Conflict of Interest

The authors declare that the research was conducted in the absence of any commercial or financial relationships that could be construed as a potential conflict of interest.

## Publisher’s Note

All claims expressed in this article are solely those of the authors and do not necessarily represent those of their affiliated organizations, or those of the publisher, the editors and the reviewers. Any product that may be evaluated in this article, or claim that may be made by its manufacturer, is not guaranteed or endorsed by the publisher.

## Supplementary Material

Supplementary Figure 1 Deep learning model development pipeline. Given the *x* as the input data of medical images, our goal is to classify these images into the corresponding Bilsky class. We first extract the region of interest (ROI) of x and feed them to the feature extractor and perturbation generator. The produced r and r_adv_ are the representation and virtual adversarial perturbation of data, respectively. We assign prototypes for each Bilsky class in the embedding space and calculate prediction probability for both the original and perturbated data points *via* a SoftMax over the negative of distance to the class prototypes. Correspondingly, ${\bar{y}}_{1}$ and ${\hat{y}}_{1}$ are the original prediction and perturbated predictions. Finally, the deep learning network is trained by minimizing the virtual adversarial loss on consistency regularization and the cross-entropy loss on the prediction probability. Note, in the embedding space, the orange-colored points are prototypes for each Bilsky class, data points of other colors represent images with different Bilsky classes. The grey-colored points are original data before perturbation.

Supplementary Figure 2 Flow chart of deep learning model deployment for clinical usage. We embed the developed deep learning model in the above pipeline for deployment. Input MRI images from patient studies will go through ROI detection with the clinicians, then the developed model is used to make predictions for the studies and report the prediction results back to the clinicians

Supplementary Table 1 MRI Platform and parameters for MRI spine axial T2-weighted Imaging. TE, echo time; TR, repetition time; GE, General Electric, *MRI scanner at the external center (Ng Teng Fong General Hospital, Singapore). All four other scanners were situated at the National University Hospital, Singapore. All studies were performed in the supine position with a torso coil.

Supplementary Table 2 Ablation study on the developed model.

Supplementary Table 3 Comparison study on the developed model.

Supplementary Table 4 Confusion matrix of the deep learning model on the internal test set.

Supplementary Table 5 Confusion matrix of the deep learning model on the external test set.
