# Supplementary material for: Deep Learning Model for Classifying Metastatic Epidural Spinal Cord Compression on MRI
Source: Front Oncol. 2022 May 4;12:849447. doi: 10.3389/fonc.2022.849447 (PMC9114468; doi:10.3389/fonc.2022.849447)
Supplement: Supplementary Figure 1 — Deep learning model development pipeline. Given the x as the input data of medical images, our goal is to classify these images into the corresponding Bilsky class. We first extract the region of interest (ROI) of x and feed them to the feature extractor and perturbation generator. The produced r and radv are the representation and virtual adversarial perturbation of data, respectively. We assign prototypes for each Bilsky class in the embedding space and calculate prediction probability for both the original and perturbated data points via a SoftMax over the negative of distance to the class prototypes. Correspondingly, y¯1 and y^1 are the original prediction and perturbated predictions. Finally, the deep learning network is trained by minimizing the virtual adversarial loss on consistency regularization and the cross-entropy loss on the prediction probability. Note, in the embedding space, the orange-colored points are prototypes for each Bilsky class, data points of other colors represent images with different Bilsky classes. The grey-colored points are original data before perturbation. [file DataSheet_1.docx]

**Supplementary Tables and Figures**

**Supplementary Table 1:**MRI Platform and parameters for MRI spine axial T2-weighted Imaging

| **Sequence** | **GE 1.5-T** | **GE 3.0-T** | **Siemens 1.5-T** | **Siemens**  **3.0-T** | **Siemens**  **1.5T*** |
| --- | --- | --- | --- | --- | --- |
| TR (msec) | 3500 | 5300 | 4000 | 5300 | 2000 |
| TE (msec) | 80 | 100 | 90 | 100 | 87 |
| Section thickness (mm) | 5 | 5 | 5 | 5 | 4 |
| Gap (mm) | 6 | 6 | 6 | 6 | 10 |
| Field of view (mm^2^) | 200 x 200 | 200 x 200 | 160 x 160 | 160 x 160 | 180 x 180 |
| Matrix | 512 x 512 | 512 x 512 | 320 x 320 | 640 x 640 | 384 x 384 |

TE = echo time, TR = repetition time, GE = General Electric, *MRI scanner at the external center (Ng Teng Fong General Hospital, Singapore). All four other scanners were situated at the National University Hospital, Singapore. All studies were performed in the supine position with a torso coil.

**Ablation Study**

We conducted an ablation study to demonstrate the effectiveness of the virtual adversarial loss and the compact regularization loss. Supplementary table 4 shows the ablation study results. In this experiment, we use average per class accuracy as the single metric to evaluate the performance of each type of model. Average per class accuracy is the average of accuracy for each class, which is widely used to evaluate deep learning models when the task has imbalanced classes. We consider four types of models in this study: (1). **Plain** is the convolutional prototypical network without either virtual adversarial loss or the compact regularization loss; (2). **Plain+Comp** is the convolutional prototypical network with compact regularization loss; (3). **Plain+VAT** is the convolutional prototypical network with virtual adversarial loss; (4). **Full** is our proposed convolutional prototypical network with both virtual adversarial loss and compact regularization loss.

**Supplementary Table 2:** Ablation study on the developed model.

| Model | Internal Test Set  Average Per Class Accuracy (%) | External Test Set  Average Per Class Accuracy (%) |
| --- | --- | --- |
| **Plain** | 90.57 | 89.39 |
| **Plain+Comp** | 93.93 | 91.89 |
| **Plain+VAT** | 93.44 | 92.62 |
| **Full** | **95.60** | **94.03** |

As shown in the table, adding compact regularization loss improves the performance of the plain model, this is because the compact regularizer draws the data points closer to their corresponding prototypes in the embedding space, which increases the margin between different class clusters and leads to more discriminative features. Furthermore, with compact regularization, the model also obtains better results on the unseen external test set. Adding the virtual adversarial loss also improves the performance of the plain model, this is because virtual adversarial loss will penalize inconsistency predictions around the neighbourhood of a data point with local perturbations and leads to smoother decision boundaries. External test performance also shows that with virtual adversarial loss, the model generalizes better to the unseen external test set. Finally, our model combines compact regularization loss and virtual adversarial loss, which achieves the best performance, indicating the complementary roles the two losses play in regularizing the network.

**Comparison Study**

To our knowledge we are the first to study automated Bilsky metastatic epidural spinal cord compression (MESCC) classification with a deep learning model. We did not find other deep learning models developed for the task in the literature. Therefore, we conducted a comparison study of our model with Resnet50 [1] and the plain Convolutional Prototypical Network (CPN) [2], which are default models scientists will try for classification tasks. Note CPN is the same **Plain** model in our ablation study (see the prior section in the supplementary material). We present the experiment results in Supplementary Table 5.

**Supplementary Table 3:** Comparison study on the developed model.

| Model | Internal Test Set  Average Per Class Accuracy (%) | External Test Set  Average Per Class Accuracy (%) |
| --- | --- | --- |
| Resnet50 [1] | 89.19 | 87.34 |
| CPN [2] | 90.57 | 89.39 |
| Ours | **95.60** | **94.03** |

As shown in the table, our specially designed model significantly outperforms existing standard solutions for the task both on the unseen internal and external test sets, which demonstrates the effectiveness and robustness of our model design.

[1]. He K, Zhang X, Ren S, Sun J. Deep residual learning for image recognition. Proceedings of the IEEE conference on computer vision and pattern recognition (2016), pp. 770-778.

[2]. Snell J, Swersky K, Zemel R. Prototypical networks for few-shot learning. Advances in neural information processing systems. 2017;30.

**Supplementary Table 4**: Confusion matrix of the deep learning model on the internal test set.

|  | Total  84+982=1066 | Predicted condition | |
| --- | --- | --- | --- |
|  |  | High-grade | Low-grade |
| Actual condition | High-grade | 82 | 2 |
|  | Low-grade | 63 | 919 |

**Supplementary Table 5**: Confusion matrix of the deep learning model on the external test set.

|  | Total  169+585=754 | Predicted condition | |
| --- | --- | --- | --- |
|  |  | High-grade | Low-grade |
| Actual condition | High-grade | 152 | 17 |
|  | Low-grade | 11 | 574 |

**
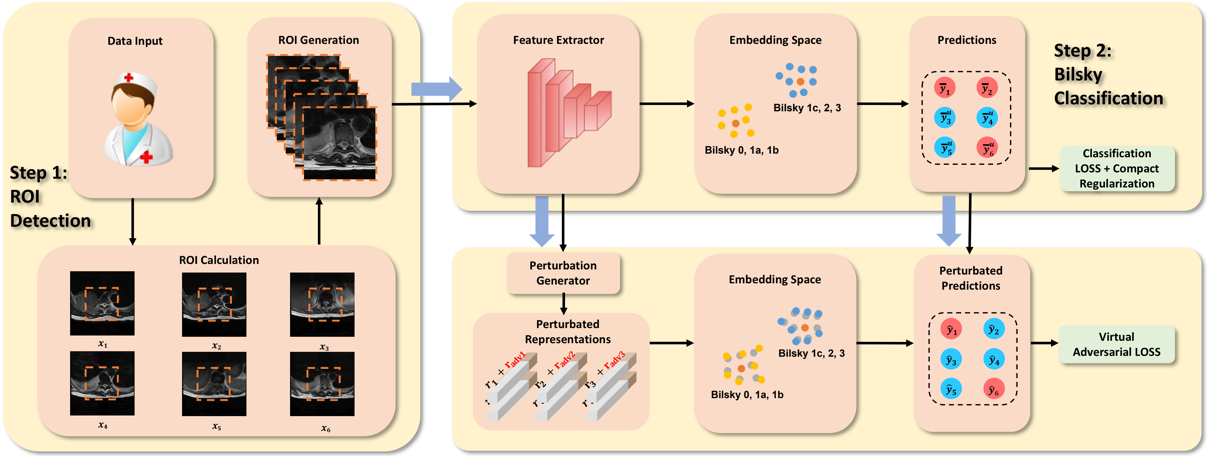
**

**Supplementary Figure 1:** Deep learning model development pipeline. Given the $\boldsymbol{x}$as the input data of medical images, our goal is to classify these images into the corresponding Bilsky class. We first extract the region of interest (ROI) of x and feed them to the feature extractor and perturbation generator. The produced $\boldsymbol{r}$and $\boldsymbol{r}_{\boldsymbol{adv}}$are the representation and virtual adversarial perturbation of data, respectively. We assign prototypes for each Bilsky class in the embedding space and calculate prediction probability for both the original and perturbated data points via a SoftMax over the negative of distance to the class prototypes. Correspondingly, ${\overline{\boldsymbol{y}}}_{\boldsymbol{1}}$and ${\hat{\boldsymbol{y}}}_{\boldsymbol{1}}$ are the original prediction and perturbated predictions. Finally, the deep learning network is trained by minimizing the virtual adversarial loss on consistency regularization and the cross-entropy loss on the prediction probability. Note, in the embedding space, the orange-colored points are prototypes for each Bilsky class, data points of other colors represent images with different Bilsky classes. The grey-colored points are original data before perturbation.

Input MRI images from patient studies

ROI detection

Deep learning model prediction

Report prediction results

**Supplementary Figure 2:** Flow chart of deep learning model deployment for clinical usage. We embed the developed deep learning model in the above pipeline for deployment. Input MRI images from patient studies will go through ROI detection with the clinicians, then the developed model is used to make predictions for the studies and report the prediction results back to the clinicians.
